# Solitary Metastasis to the Breast From Thigh Myxoid Liposarcoma

**DOI:** 10.7759/cureus.45559

**Published:** 2023-09-19

**Authors:** Thuy-Linh Tran, Irene Tsai, Hyung Won Choi

**Affiliations:** 1 School of Medicine, University of California, Irvine, Orange, USA; 2 Department of Radiological Sciences, University of California, Irvine, Orange, USA

**Keywords:** breast mass, ultrasound, mammography, myxoid liposarcoma, breast metastasis

## Abstract

Metastasis to the breast from non-mammary malignancies are rare and suggestive of advanced disease. Accurate and prompt diagnosis of breast metastasis can provide important prognostic information and guide treatment planning. Interestingly, in contrast to primary breast malignancies, non-mammary metastatic breast lesions often have benign-appearing imaging characteristics. Knowing a patient's clinical history and having prior breast imaging studies for comparison is important for making accurate assessments and appropriate recommendations. Imaging-guided biopsy is often indicated for definitive tissue diagnosis. We report a rare case of solitary metastasis to the breast from thigh myxoid liposarcoma.

## Introduction

Metastasis to the breast from non-mammary malignancies is rare, accounting for approximately 2% of all breast malignancies [1,2]. Some postulate that the rarity of metastasis to the breast is secondary to the dominant presence of fibrous tissue and relatively poor blood supply in the breast [3]. Although exceedingly rare, the presence of breast metastasis provides important prognostic information, as it is not uncommonly indicative of advanced disease and can affect treatment planning.

Common non-mammary sites of origin for breast metastasis include skin, lung, ovary, and hematologic sites [1,2]. Breast metastasis from soft tissue sarcoma is relatively rarer in comparison. Here, we report an exceptionally rare case of solitary breast metastasis from thigh myxoid liposarcoma. Only two additional case reports of solitary breast metastasis of myxoid liposarcoma could be confirmed in our search of medical literature [4,5]. Several more cases of breast metastasis from myxoid liposarcoma have been noted in other studies; however, these studies do not include individual clinical information or specify if the breast metastasis was solitary or part of a disseminated disease [6-8].

## Case presentation

**Figure 1:**
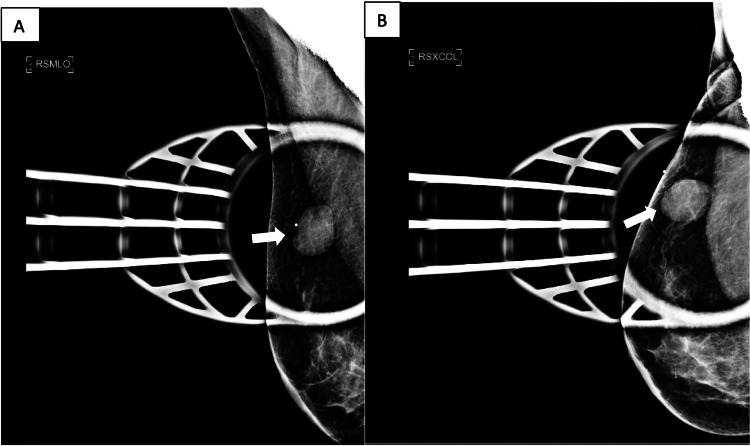
Diagnostic mammogram of the right breast (A) Mediolateral oblique spot compression and (B) exaggerated craniocaudal spot compression views demonstrate a 27 mm circumscribed oval mass (white arrow) in the upper-outer quadrant of the right breast at a posterior depth. The mass corresponds to the skin BB marker denoting the patient’s area of palpable abnormality.

**Figure 2:**
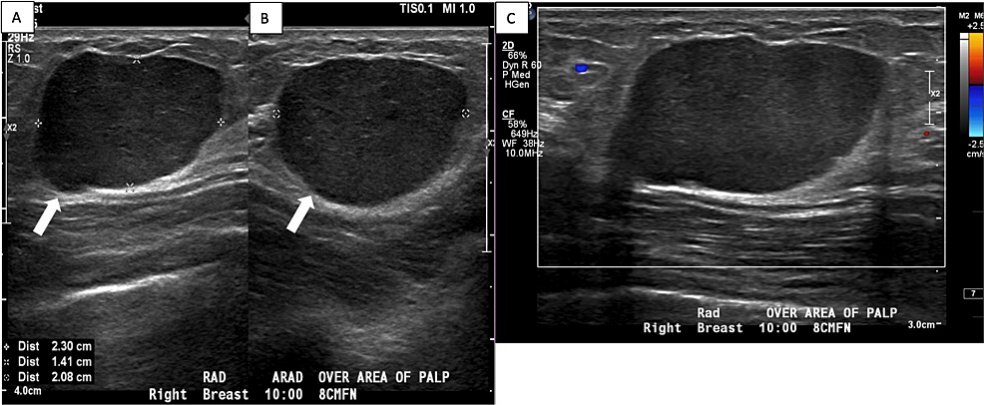
Targeted sonographic images of the right breast Grayscale radial (A) and anti-radial (B) images of the right breast in the patient’s area of concern at 10 o’clock, 8 cm from the nipple, demonstrate a circumscribed oval hypoechoic mass (white arrow), measuring 23 mm. Its oval shape, circumscribed margin, and parallel orientation are characteristic of a BI-RADS Category 3 lesion. (C) Color Doppler image of the mass shows no significant associated vascularity within or around the mass. BI-RADS: Breast Imaging Reporting and Data System

A 53-year-old female presented with a new, enlarging, right breast mass five months after her diagnosis of left thigh myxoid liposarcoma. The patient reported a family history of breast cancer (mother and maternal aunt). A diagnostic mammogram showed a 27 mm oval mass with circumscribed margins in the upper-outer quadrant of the right breast at a posterior depth, correlating to the patient’s palpable abnormality (Figure 1). The right breast mass was not present on her mammogram from 18 months prior. Ultrasound demonstrated a corresponding oval hypoechoic mass with circumscribed margins measuring 23 mm in the right breast at 10 o’clock, located 8 cm from the nipple (Figure 2). No additional solid or cystic mass was seen in either breast. No suspicious axillary lymph nodes were seen.

**Figure 3:**
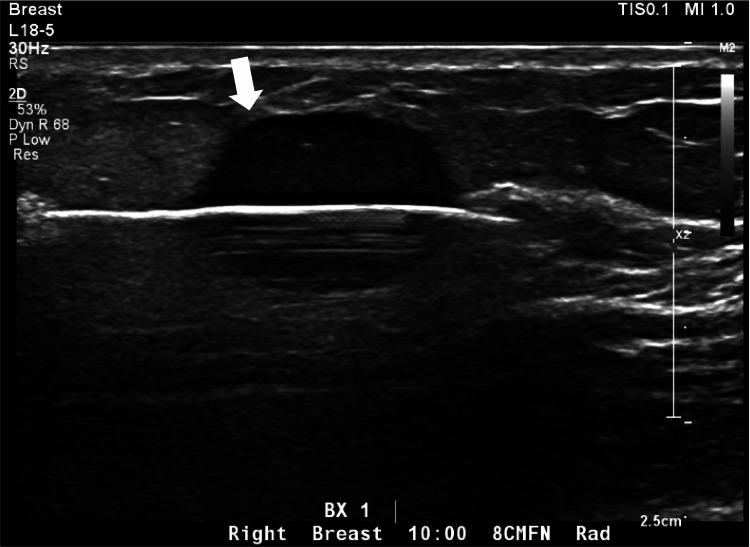
Ultrasound-guided core needle biopsy of the mass. The postfire grayscale image shows the successful passage of the biopsy needle through the target mass (white arrow).

**Figure 4:**
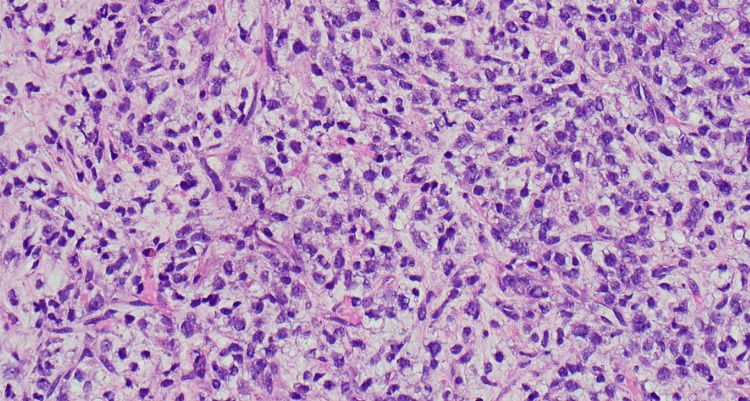
H&E stain from the biopsy of the mass in the right breast Lipoblasts dispersed through the myxoid matrix H&E: hematoxylin and eosin

Although the breast mass demonstrated relatively benign-appearing imaging features that are often seen with Breast Imaging Reporting and Data System (BI-RADS) Category 3, given it was a new, enlarging mass in a postmenopausal woman with a known history of malignancy and a family history of breast cancer, a BI-RADS Category 4 was assigned with recommendation for definitive tissue diagnosis via ultrasound-guided core needle biopsy [9]. The tissue specimen from the ultrasound-guided biopsy (Figure 3) showed liposarcoma with high-grade round cell components in the majority of the tissue (Figure 4). Rearrangement of DDIT3 (DNA Damage Inducible Transcript 3), a transcription factor associated with myxoid liposarcoma, was detected by fluorescence in situ hybridization (FISH), supporting the diagnosis.

The positron emission tomography and computed tomography (PET/CT) examination performed at another imaging facility approximately three months prior to the breast diagnostic examination showed no lesions suspicious for distant metastasis. Wide local excision of the right breast mass was performed at the time of the thigh mass removal. The negative margins of the excised breast mass were confirmed intra-operatively. Postoperative pathology of the breast mass showed grade 2 myxoid liposarcoma, measuring 2.6 cm x 2.4 cm x 1.9 cm, similar to what was seen on diagnostic imaging. The immediate postoperative course was complicated by a breast seroma that was drained for symptom alleviation. Given that negative margins were obtained for both the breast and lower extremity liposarcomas and the postoperative PET/CT examination did not show any suspicious lesions, the decision was made for surveillance with routine imaging.

## Discussion

Myxoid liposarcoma (MLS) is the second most common histologic subtype of liposarcoma [4]. Other subtypes of liposarcoma include well-differentiated, dedifferentiated, round-cell, and pleomorphic, with the differentiated subtype being the most common [10,11]. MLS is unique from other types of sarcomas in that it tends to metastasize to extrapulmonary sites such as soft tissue, retroperitoneum, and bone. This can imply a more favorable prognosis for the patient, as studies have shown that patients with extrapulmonary metastasis have a longer disease-free interval compared to patients with pulmonary metastasis [8].

Due to the rarity of solitary breast metastasis of MLS, there has been no published consensus on its standard treatment. Given the relatively more favorable prognosis of patients with extrapulmonary MLS metastasis, some suggest that treatment of solitary breast metastasis should be aggressive and curative with wide local surgical excision [4,12]. Due to the paucity of data, no formal conclusions on the efficacy or benefits of wide local surgical excision have been reached [13,14]. Some studies have shown that MLS has a higher sensitivity to radiotherapy compared to other soft tissue sarcoma subtypes [10,15].

The distinction between secondary breast metastasis and primary breast malignancy is important, as they differ in the management and treatment approach. Interestingly, a significant number of secondary breast metastases initially present as palpable breast lumps in patients with no known history of malignancy [14]. Knowing the clinical history, such as the patient’s age and history of cancer, family history of cancer, and comparison to prior breast imaging studies, are thus very important for arriving at an accurate assessment and recommendation. Often, imaging-guided biopsy for definitive histologic diagnosis is indicated for indeterminate breast lesions to guide subsequent workup and treatment planning.

Metastatic non-mammary breast lesions tend to differ in their imaging appearance compared to primary breast cancer. Unilateral breast involvement is more commonly observed for metastatic non-mammary lesions, rather than multiple bilateral masses. Many non-mammary breast lesions can have a deceptively benign appearance on breast imaging, commonly presenting as a hypoechoic oval or round mass with circumscribed margins, which are typically seen for BI-RADS Category 3 lesions [9,16]. Thus, they can be mistaken for benign breast lesions, such as fibroadenomas, however, careful consideration of clinical history, such as patient's age, history of malignancy, and comparison to prior imaging, can help appropriately raise suspicion for malignancy. Some metastasis, such as sarcoma metastasis, may sometimes present as a hyperechoic mass on ultrasound. Certain breast metastasis, such as those of ovarian or thyroid origin, can have associated calcifications.

A distinctive imaging feature of non-mammary breast metastasis is the lack of spiculated margins due to the lack of desmoplastic reaction that is commonly seen in primary breast malignancy. Also, breast metastasis from a non-mammary origin commonly presents in subcutaneous tissue rather than parenchymal breast tissue [1]. It is usually not associated with skin or nipple retraction [17]. In histologic sections, a sharp transition at the lesion border is often seen, with no significant stromal elastosis, which explains the benign imaging appearance of breast metastasis from non-mammary origin.

## Conclusions

Solitary breast metastasis of myxoid liposarcoma is very rare and only limited information is available regarding its imaging appearance and treatment. Based on the small number of cases reported so far in the literature, breast metastasis from myxoid liposarcoma tends to present with benign-appearing imaging features that overlap with benign breast lesions. Knowing the patient’s clinical history is essential for appropriate assessment and management. A definitive tissue diagnosis with core needle biopsy is often indicated, as a diagnosis of breast metastasis can change a patient’s prognosis and treatment planning.
